# Supplementary material for: Sex-specific associations of social determinants of health and genetic risk factors with atherosclerotic cardiovascular diseases incidence in the general population
Source: Am J Prev Cardiol. 2026 Feb 25;29:101505. doi: 10.1016/j.ajpc.2026.101505 (PMC13329579; doi:10.1016/j.ajpc.2026.101505)
Supplement: Supplementary file 1 [file mmc1.docx]

**SUPPLEMENTAL DATA**

**Sex-specific associations of social determinants of health and genetic risk factors with atherosclerotic cardiovascular diseases incidence in the general population**

Audrey Paulin^a^, Louis-Jacques Ruel^a^, Sébastien Thériault^a,b^, and Benoit J. Arsenault^a,c^

1. Centre de recherche de l’Institut universitaire de cardiologie et de pneumologie de Québec – Université Laval, Québec (QC), Canada
2. Department of Molecular Biology, Medical Biochemistry and Pathology, Faculty of Medicine, Université Laval, Québec (QC), Canada
3. Department of Medicine, Faculty of Medicine, Université Laval, Québec (QC), Canada

*Address for correspondence

Benoit Arsenault, PhD

Centre de recherche de l’Institut universitaire de cardiologie et de pneumologie de Québec – Université Laval

Y-3106, Pavillon Marguerite D'Youville, 2725 chemin Ste-Foy

Québec (QC), Canada G1V 4G5

Telephone: 418-656-8711 ext. 3498

Email: benoit.arsenault@criucpq.ulaval.ca

**Supplemental Table 1.** Distribution of the individual PSS variables across the PSS categories presented by sex.

| **Factor** | **Females** | | | | **Males** | | | |
| --- | --- | --- | --- | --- | --- | --- | --- | --- |
|  | **Total** | **Low PSS** | **Moderate PSS** | **High PSS** | **Total** | **Low PSS** | **Moderate PSS** | **High PSS** |
| N | 179900 | 60173 | 83169 | 36558 | 141116 | 47974 | 66201 | 26941 |
| **SOCIOECONOMIC FACTORS** |  |  |  |  |  |  |  |  |
| **Average total household income before tax** |  |  |  |  |  |  |  |  |
| Less than 18,000£ | 36287 (20.2%) | 4156 (6.9%) | 10913 (13.1%) | 21218 (58%) | 24258 (17.2%) | 2240 (4.7%) | 6528 (9.9%) | 15490 (57.5%) |
| 18,000£ to 30,999£ | 39704 (22.1%) | 13171 (21.9%) | 19257 (23.2%) | 7276 (19.9%) | 30881 (21.9%) | 9574 (20%) | 14734 (22.3%) | 6573 (24.4%) |
| 31,000£ to 51,999£ | 38566 (21.4%) | 17195 (28.6%) | 20187 (24.3%) | 1184 (3.2%) | 34975 (24.8%) | 14692 (30.6%) | 19113 (28.9%) | 1170 (4.3%) |
| 52,000£ to 100,000£ | 28461 (15.8%) | 13399 (22.3%) | 15046 (18.1%) | 16 (0%) | 29373 (20.8%) | 13412 (28%) | 15943 (24.1%) | 18 (0.1%) |
| Greater than 100,000£ | 7257 (4%) | 3792 (6.3%) | 3465 (4.2%) | 0 (0%) | 7971 (5.6%) | 4062 (8.5%) | 3909 (5.9%) | 0 (0%) |
| Do not know, prefer not to answer or unavailable | 29625 (16.5%) | 8460 (14.1%) | 14301 (17.2%) | 6864 (18.8%) | 13658 (9.7%) | 3994 (8.3%) | 5974 (9%) | 3690 (13.7%) |
| **Education level** |  |  |  |  |  |  |  |  |
| College or University degree | 56281 (31.3%) | 27035 (44.9%) | 25742 (31%) | 3504 (9.6%) | 48580 (34.4%) | 22525 (47%) | 23293 (35.2%) | 2762 (10.3%) |
| Other professional qualifications | 27642 (15.4%) | 9436 (15.7%) | 14783 (17.8%) | 3423 (9.4%) | 19854 (14.1%) | 6627 (13.8%) | 11052 (16.7%) | 2175 (8.1%) |
| NVQ or HND or HNC or equivalent | 18839 (10.5%) | 5387 (9%) | 8655 (10.4%) | 4797 (13.1%) | 21958 (15.6%) | 6542 (13.6%) | 11261 (17%) | 4155 (15.4%) |
| A levels/AS levels or equivalent | 10516 (5.8%) | 3951 (6.6%) | 5094 (6.1%) | 1471 (4%) | 7247 (5.1%) | 2829 (5.9%) | 3302 (5%) | 1116 (4.1%) |
| O levels/GCSEs or equivalent or CSEs or equivalent | 35421 (19.7%) | 10363 (17.2%) | 17511 (21.1%) | 7547 (20.6%) | 19326 (13.7%) | 6070 (12.7%) | 9017 (13.6%) | 4239 (15.7%) |
| None of the above | 29661 (16.5%) | 3598 (6%) | 10750 (12.9%) | 15313 (41.9%) | 22934 (16.3%) | 3035 (6.3%) | 7801 (11.8%) | 12098 (44.9%) |
| Prefer not to answer or unavailable | 1540 (0.9%) | 403 (0.7%) | 634 (0.8%) | 503 (1.4%) | 1217 (0.9%) | 346 (0.7%) | 475 (0.7%) | 396 (1.5%) |
| **Education score** |  |  |  |  |  |  |  |  |
| Above median | 85398 (47.5%) | 21780 (36.2%) | 31228 (37.5%) | 32390 (88.6%) | 68239 (48.4%) | 18605 (38.8%) | 25675 (38.8%) | 23959 (88.9%) |
| Below median | 90181 (50.1%) | 37150 (61.7%) | 49807 (59.9%) | 3224 (8.8%) | 69225 (49.1%) | 28240 (58.9%) | 38816 (58.6%) | 2169 (8.1%) |
| Unavailable | 4321 (2.4%) | 1243 (2.1%) | 2134 (2.6%) | 944 (2.6%) | 3652 (2.6%) | 1129 (2.4%) | 1710 (2.6%) | 813 (3%) |
| **Current employment status** |  |  |  |  |  |  |  |  |
| In paid employment or self-employed; Retired; Doing unpaid or voluntary work; Full or part-time student | 165506 (92%) | 56833 (94.4%) | 78062 (93.9%) | 30611 (83.7%) | 131918 (93.5%) | 46738 (97.4%) | 64351 (97.2%) | 20829 (77.3%) |
| Looking after home and/or family; Unable to work because of sickness or disability; Unemployed; None of the above | 13855 (7.7%) | 3166 (5.3%) | 4900 (5.9%) | 5789 (15.8%) | 8753 (6.2%) | 1086 (2.3%) | 1689 (2.6%) | 5978 (22.2%) |
| Prefer not to answer or unavailable | 539 (0.3%) | 174 (0.3%) | 207 (0.2%) | 158 (0.4%) | 445 (0.3%) | 150 (0.3%) | 161 (0.2%) | 134 (0.5%) |
| **PSYCHOSOCIAL FACTORS** |  |  |  |  |  |  |  |  |
| **Number of people in the household** |  |  |  |  |  |  |  |  |
| Living alone | 34759 (19.3%) | 8983 (14.9%) | 10968 (13.2%) | 14808 (40.5%) | 23342 (16.5%) | 5771 (12%) | 6377 (9.6%) | 11194 (41.6%) |
| Not living alone | 144159 (80.1%) | 50868 (84.5%) | 71966 (86.5%) | 21325 (58.3%) | 117009 (82.9%) | 41984 (87.5%) | 59663 (90.1%) | 15362 (57%) |
| Do not know, prefer not to answer or unavailable | 982 (0.5%) | 322 (0.5%) | 235 (0.3%) | 425 (1.2%) | 765 (0.5%) | 219 (0.5%) | 161 (0.2%) | 385 (1.4%) |
| **How often are you able to confide in someone close to you?** |  |  |  |  |  |  |  |  |
| Never or almost never | 18923 (10.5%) | 4616 (7.7%) | 7957 (9.6%) | 6350 (17.4%) | 25380 (18%) | 6897 (14.4%) | 10591 (16%) | 7892 (29.3%) |
| Once every few months | 9624 (5.3%) | 2951 (4.9%) | 4294 (5.2%) | 2379 (6.5%) | 7483 (5.3%) | 2597 (5.4%) | 3212 (4.9%) | 1674 (6.2%) |
| About once a month | 10076 (5.6%) | 3384 (5.6%) | 4388 (5.3%) | 2304 (6.3%) | 6544 (4.6%) | 2413 (5%) | 2661 (4%) | 1470 (5.5%) |
| About once a week | 22143 (12.3%) | 7302 (12.1%) | 9437 (11.3%) | 5404 (14.8%) | 12400 (8.8%) | 4405 (9.2%) | 5153 (7.8%) | 2842 (10.5%) |
| 2-3 times a week | 20119 (11.2%) | 7250 (12%) | 8812 (10.6%) | 4057 (11.1%) | 10417 (7.4%) | 3970 (8.3%) | 4500 (6.8%) | 1947 (7.2%) |
| Almost daily | 94374 (52.5%) | 33284 (55.3%) | 46264 (55.6%) | 14826 (40.6%) | 74617 (52.9%) | 26337 (54.9%) | 38334 (57.9%) | 9946 (36.9%) |
| Do not know, prefer not to answer or unavailable | 4641 (2.6%) | 1386 (2.3%) | 2017 (2.4%) | 1238 (3.4%) | 4275 (3%) | 1355 (2.8%) | 1750 (2.6%) | 1170 (4.3%) |
| **Number of social activities** |  |  |  |  |  |  |  |  |
| No social activities | 53839 (29.9%) | 16077 (26.7%) | 23459 (28.2%) | 14303 (39.1%) | 41669 (29.5%) | 12983 (27.1%) | 19150 (28.9%) | 9536 (35.4%) |
| At least 1 social activities | 125582 (69.8%) | 43946 (73%) | 59514 (71.6%) | 22122 (60.5%) | 99125 (70.2%) | 34886 (72.7%) | 46939 (70.9%) | 17300 (64.2%) |
| Prefer not to answer or unavailable | 479 (0.3%) | 150 (0.2%) | 196 (0.2%) | 133 (0.4%) | 322 (0.2%) | 105 (0.2%) | 112 (0.2%) | 105 (0.4%) |
| **Frequency of friend/family visits** |  |  |  |  |  |  |  |  |
| Almost daily | 25539 (14.2%) | 6342 (10.5%) | 11277 (13.6%) | 7920 (21.7%) | 12189 (8.6%) | 2975 (6.2%) | 5278 (8%) | 3936 (14.6%) |
| 2-4 times a week | 61631 (34.3%) | 19965 (33.2%) | 29050 (34.9%) | 12616 (34.5%) | 37962 (26.9%) | 12141 (25.3%) | 18379 (27.8%) | 7442 (27.6%) |
| About once a week | 60777 (33.8%) | 22052 (36.6%) | 28513 (34.3%) | 10212 (27.9%) | 53646 (38%) | 19353 (40.3%) | 25960 (39.2%) | 8333 (30.9%) |
| About once a month | 20160 (11.2%) | 7878 (13.1%) | 9476 (11.4%) | 2806 (7.7%) | 22160 (15.7%) | 8603 (17.9%) | 10582 (16%) | 2975 (11%) |
| Once every few months | 9363 (5.2%) | 3373 (5.6%) | 4126 (5%) | 1864 (5.1%) | 11259 (8%) | 4046 (8.4%) | 4927 (7.4%) | 2286 (8.5%) |
| Never or almost never | 1484 (0.8%) | 329 (0.5%) | 422 (0.5%) | 733 (2%) | 2888 (2%) | 628 (1.3%) | 822 (1.2%) | 1438 (5.3%) |
| No friends/family outside household | 308 (0.2%) | 30 (0%) | 60 (0.1%) | 218 (0.6%) | 397 (0.3%) | 44 (0.1%) | 57 (0.1%) | 296 (1.1%) |
| Do not know, prefer not to answer or unavailable | 638 (0.4%) | 204 (0.3%) | 245 (0.3%) | 189 (0.5%) | 615 (0.4%) | 184 (0.4%) | 196 (0.3%) | 235 (0.9%) |
| **Emotional distress** |  |  |  |  |  |  |  |  |
| No stressful event | 97509 (54.2%) | 33397 (55.5%) | 48006 (57.7%) | 16106 (44.1%) | 81488 (57.7%) | 28504 (59.4%) | 40712 (61.5%) | 12272 (45.6%) |
| At least 1 stressful event | 81468 (45.3%) | 26557 (44.1%) | 34850 (41.9%) | 20061 (54.9%) | 59074 (41.9%) | 19333 (40.3%) | 25341 (38.3%) | 14400 (53.5%) |
| Prefer not to answer or unavailable | 923 (0.5%) | 219 (0.4%) | 313 (0.4%) | 391 (1.1%) | 554 (0.4%) | 137 (0.3%) | 148 (0.2%) | 269 (1%) |
| **Diagnosed psychiatric disorder** |  |  |  |  |  |  |  |  |
| Yes | 44168 (24.6%) | 15744 (26.2%) | 19262 (23.2%) | 9162 (25.1%) | 22502 (15.9%) | 8136 (17%) | 9525 (14.4%) | 4841 (18%) |
| No | 135732 (75.4%) | 44429 (73.8%) | 63907 (76.8%) | 27396 (74.9%) | 118614 (84.1%) | 39838 (83%) | 56676 (85.6%) | 22100 (82%) |
| **NEIGHBOURHOOD AND LIVING ENVIRONMENT** |  |  |  |  |  |  |  |  |
| **Townsend deprivation index** |  |  |  |  |  |  |  |  |
| Above median | 87405 (48.6%) | 33528 (55.7%) | 20535 (24.7%) | 33342 (91.2%) | 68364 (48.4%) | 26969 (56.2%) | 16516 (24.9%) | 24879 (92.3%) |
| Below median | 92290 (51.3%) | 26588 (44.2%) | 62536 (75.2%) | 3166 (8.7%) | 72585 (51.4%) | 20951 (43.7%) | 49607 (74.9%) | 2027 (7.5%) |
| Unavailable | 205 (0.1%) | 57 (0.1%) | 98 (0.1%) | 50 (0.1%) | 167 (0.1%) | 54 (0.1%) | 78 (0.1%) | 35 (0.1%) |
| **Crime score** |  |  |  |  |  |  |  |  |
| Above median | 86129 (47.9%) | 33474 (55.6%) | 22997 (27.7%) | 29658 (81.1%) | 67665 (47.9%) | 26954 (56.2%) | 18620 (28.1%) | 22091 (82%) |
| Below median | 88519 (49.2%) | 25236 (41.9%) | 57360 (69%) | 5923 (16.2%) | 69122 (49%) | 19739 (41.1%) | 45369 (68.5%) | 4014 (14.9%) |
| Unavailable | 5252 (2.9%) | 1463 (2.4%) | 2812 (3.4%) | 977 (2.7%) | 4329 (3.1%) | 1281 (2.7%) | 2212 (3.3%) | 836 (3.1%) |
| **Accommodation quality** |  |  |  |  |  |  |  |  |
| Above median | 86986 (48.4%) | 29096 (48.4%) | 38811 (46.7%) | 19079 (52.2%) | 68070 (48.2%) | 23006 (48%) | 30991 (46.8%) | 14073 (52.2%) |
| Below median | 88593 (49.2%) | 29834 (49.6%) | 42224 (50.8%) | 16535 (45.2%) | 69394 (49.2%) | 23839 (49.7%) | 33500 (50.6%) | 12055 (44.7%) |
| Unavailable | 4321 (2.4%) | 1243 (2.1%) | 2134 (2.6%) | 944 (2.6%) | 3652 (2.6%) | 1129 (2.4%) | 1710 (2.6%) | 813 (3%) |
| **Type of accommodation** |  |  |  |  |  |  |  |  |
| Own outright (by you or someone in your household) | 97046 (53.9%) | 29782 (49.5%) | 49389 (59.4%) | 17875 (48.9%) | 71101 (50.4%) | 22449 (46.8%) | 36758 (55.5%) | 11894 (44.1%) |
| Own with a mortgage | 64882 (36.1%) | 27536 (45.8%) | 31146 (37.4%) | 6200 (17%) | 54324 (38.5%) | 22743 (47.4%) | 27271 (41.2%) | 4310 (16%) |
| Rent - from local authority, local council, housing association | 9399 (5.2%) | 202 (0.3%) | 213 (0.3%) | 8984 (24.6%) | 7541 (5.3%) | 144 (0.3%) | 144 (0.2%) | 7253 (26.9%) |
| Rent - from private landlord or letting agency | 4746 (2.6%) | 1388 (2.3%) | 1170 (1.4%) | 2188 (6%) | 4764 (3.4%) | 1499 (3.1%) | 1005 (1.5%) | 2260 (8.4%) |
| Pay part rent and part mortgage (shared ownership) | 531 (0.3%) | 169 (0.3%) | 99 (0.1%) | 263 (0.7%) | 376 (0.3%) | 126 (0.3%) | 70 (0.1%) | 180 (0.7%) |
| Live in accommodation rent free | 1051 (0.6%) | 366 (0.6%) | 385 (0.5%) | 300 (0.8%) | 1143 (0.8%) | 421 (0.9%) | 382 (0.6%) | 340 (1.3%) |
| None of the above | 725 (0.4%) | 239 (0.4%) | 223 (0.3%) | 263 (0.7%) | 739 (0.5%) | 241 (0.5%) | 215 (0.3%) | 283 (1.1%) |
| Prefer not to answer or unavailable | 1520 (0.8%) | 491 (0.8%) | 544 (0.7%) | 485 (1.3%) | 1128 (0.8%) | 351 (0.7%) | 356 (0.5%) | 421 (1.6%) |
| **Proximity of greenspace** |  |  |  |  |  |  |  |  |
| Above median | 76600 (42.6%) | 50988 (84.7%) | 3698 (4.4%) | 21914 (59.9%) | 60187 (42.7%) | 40719 (84.9%) | 2941 (4.4%) | 16527 (61.3%) |
| Below median | 80188 (44.6%) | 343 (0.6%) | 70653 (85%) | 9192 (25.1%) | 63170 (44.8%) | 329 (0.7%) | 56273 (85%) | 6568 (24.4%) |
| Unavailable | 23112 (12.8%) | 8842 (14.7%) | 8818 (10.6%) | 5452 (14.9%) | 17759 (12.6%) | 6926 (14.4%) | 6987 (10.6%) | 3846 (14.3%) |
| **Proximity of bluespace** |  |  |  |  |  |  |  |  |
| Above median | 78570 (43.7%) | 30306 (50.4%) | 32103 (38.6%) | 16161 (44.2%) | 61030 (43.2%) | 23642 (49.3%) | 25662 (38.8%) | 11726 (43.5%) |
| Below median | 78218 (43.5%) | 21025 (34.9%) | 42248 (50.8%) | 14945 (40.9%) | 62327 (44.2%) | 17406 (36.3%) | 33552 (50.7%) | 11369 (42.2%) |
| Unavailable | 23112 (12.8%) | 8842 (14.7%) | 8818 (10.6%) | 5452 (14.9%) | 17759 (12.6%) | 6926 (14.4%) | 6987 (10.6%) | 3846 (14.3%) |
| **Proximity of natural environment** |  |  |  |  |  |  |  |  |
| Above median | 87277 (48.5%) | 59072 (98.2%) | 2383 (2.9%) | 25822 (70.6%) | 68170 (48.3%) | 46963 (97.9%) | 1932 (2.9%) | 19275 (71.5%) |
| Below median | 91037 (50.6%) | 493 (0.8%) | 80204 (96.4%) | 10340 (28.3%) | 71431 (50.6%) | 405 (0.8%) | 63749 (96.3%) | 7277 (27%) |
| Unavailable | 1586 (0.9%) | 608 (1%) | 582 (0.7%) | 396 (1.1%) | 1515 (1.1%) | 606 (1.3%) | 520 (0.8%) | 389 (1.4%) |

**
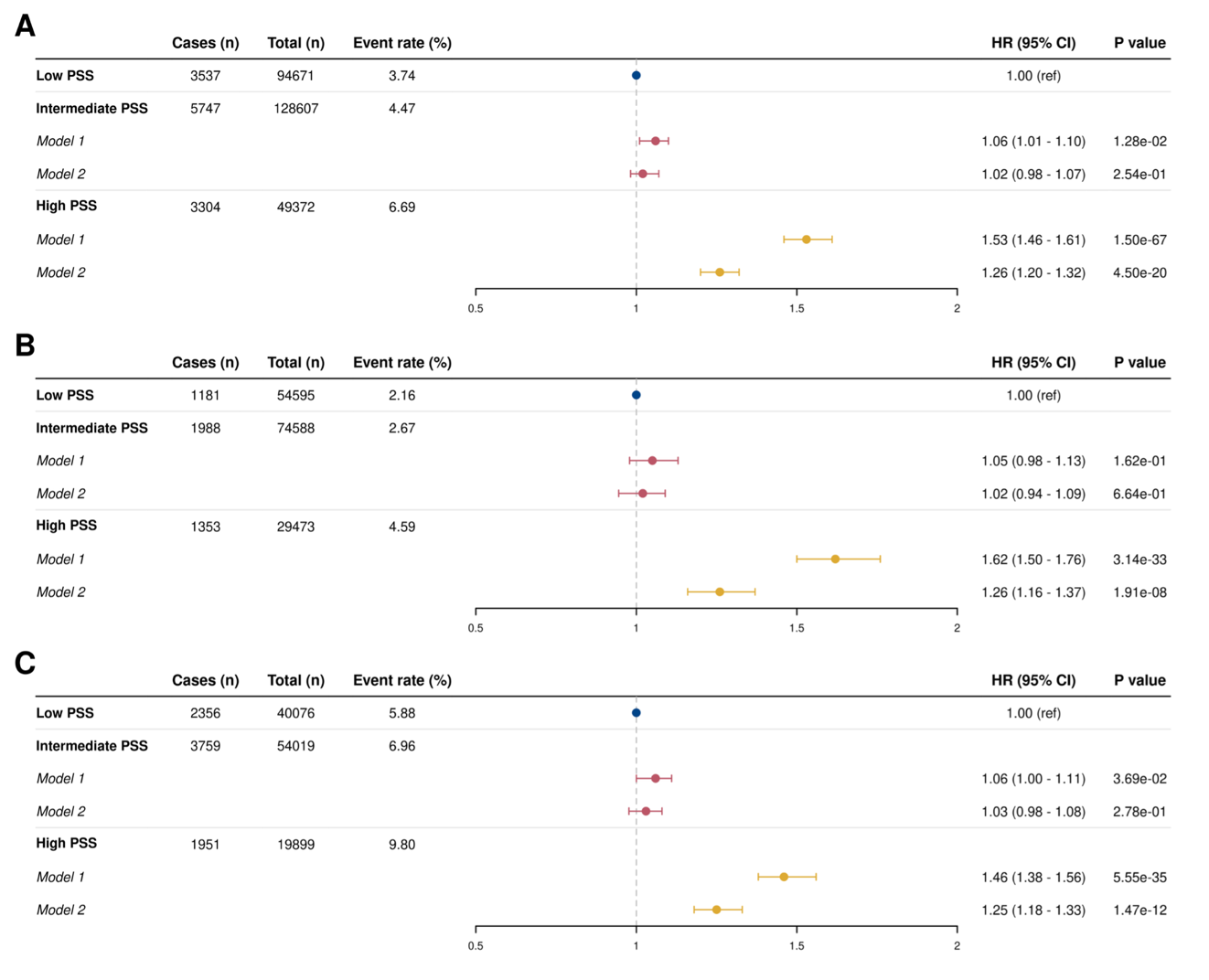
**

**Supplemental Figure 1.** Impact of a polysocial risk score (PSS) on the incidence of atherosclerotic cardiovascular diseases excluding participants treated with statins. Results are presented in: A) all participants, B) females and C) males. Model 1 is adjusted for age, sex, and ethnicity while model 2 is adjusted for age, sex, ethnicity, and components of a cardiovascular health score.

**
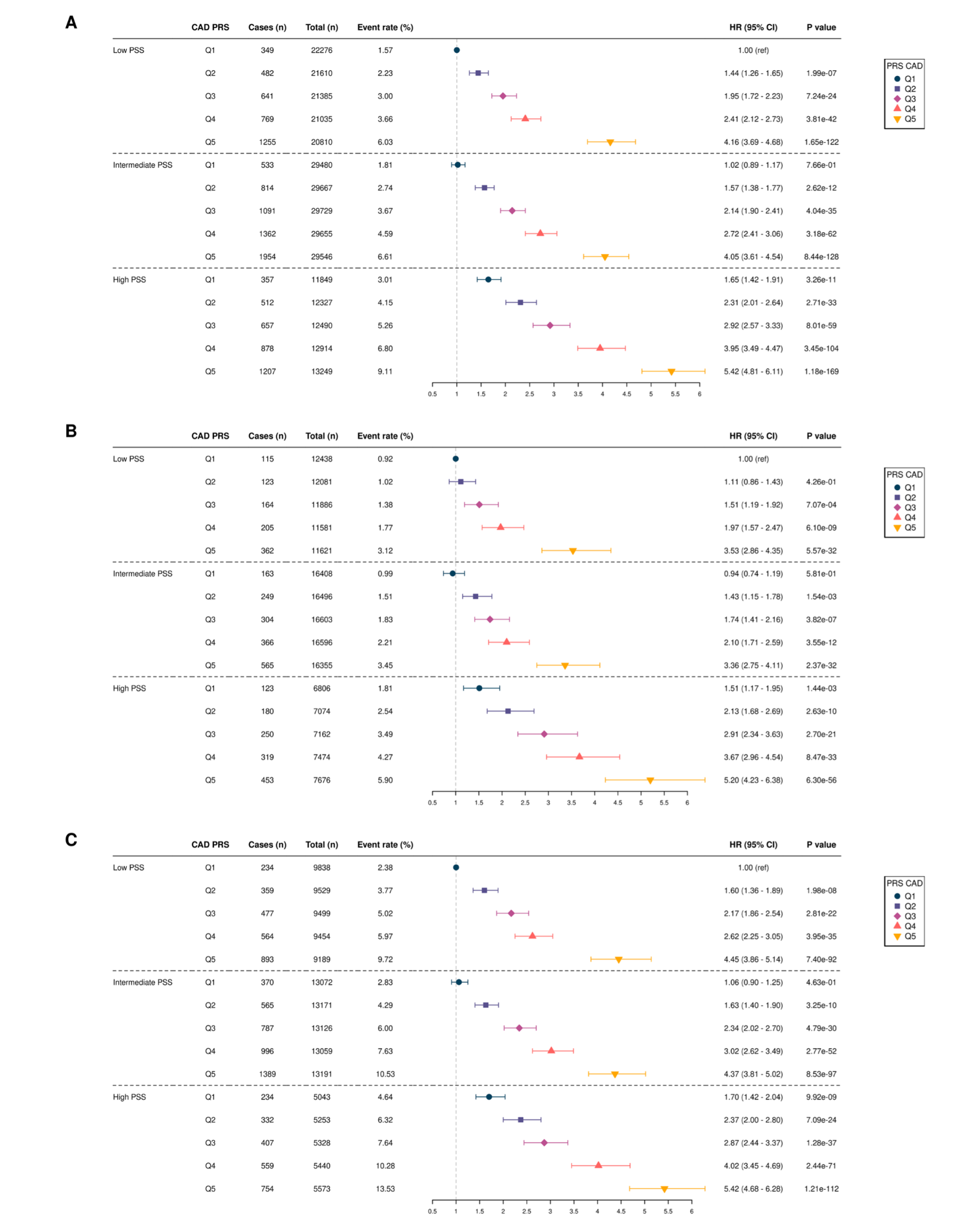
**

**Supplemental Figure 2.** Joint contributions of a polysocial risk score (PSS) and quintiles of a polygenic risk score for coronary artery disease (CAD-PRS) on the incidence of CAD. Results are presented in: A) all participants, B) females, and C) males. Cox regression analyses are adjusted for age, sex, and ethnicity.

**
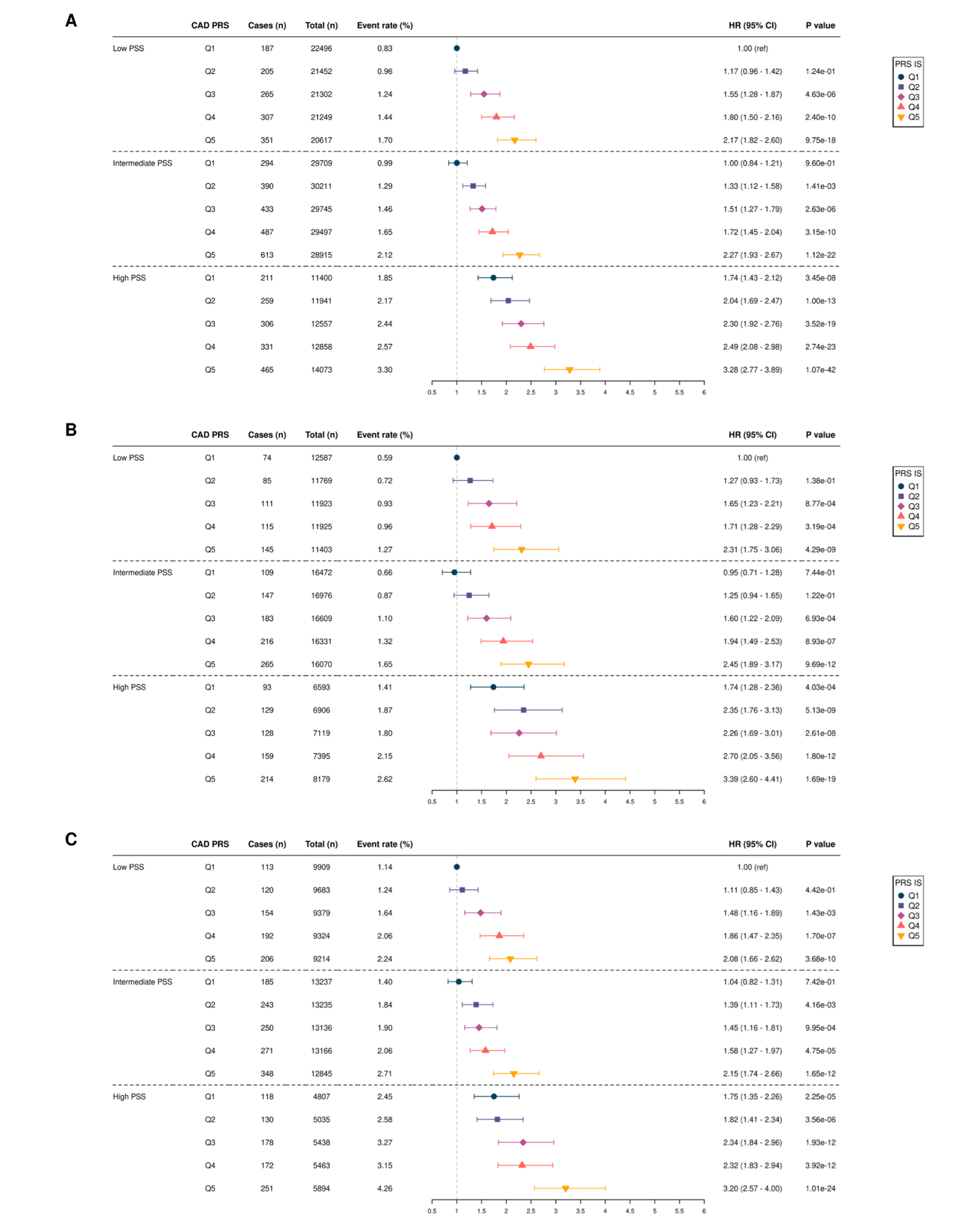
**

**Supplemental Figure 3.** Joint contributions of a polysocial risk score (PSS) and quintiles of a polygenic risk score for ischemic stroke (IS-PRS) on the incidence of IS. Results are presented in: A) all participants, B) females, and C) males. Cox regression analyses are adjusted for age, sex, and ethnicity.
